# Precise-Integration Time-Domain Formulation for Optical Periodic Media

**DOI:** 10.3390/ma14247896

**Published:** 2021-12-20

**Authors:** Joan Josep Sirvent-Verdú, Jorge Francés, Andrés Márquez, Cristian Neipp, Mariela Álvarez, Daniel Puerto, Sergi Gallego, Inmaculada Pascual

**Affiliations:** 1 Departamento de Física, Ingeniería de Sistemas y Teoría de la Señal, Universidad de Alicante, P.O. Box 99, 03080 Alicante, Spain; jjsv2@gcloud.ua.es (J.J.S.-V.); andres.marquez@ua.es (A.M.); cristian@ua.es (C.N.); mariela.alvarez@ua.es (M.Á.); dan.puerto@ua.es (D.P.); sergi.gallego@ua.es (S.G.); 2 I.U. Física Aplicada a las Ciencias y las Tecnologías, Universidad de Alicante, P.O. Box 99, 03080 Alicante, Spain; pascual@ua.es; 3 Departamento de Óptica, Anatomía y Farmacología, Universidad de Alicante, P.O. Box 99, 03080 Alicante, Spain

**Keywords:** computational electromagnetics, precise-integration time-domain (PITD) method, periodic media, anisotropic media, diffractive optics, 42.40.Eq, 47.11.Bc, 42.70.Jk, 78A10

## Abstract

A numerical formulation based on the precise-integration time-domain (PITD) method for simulating periodic media is extended for overcoming the Courant-Friedrich-Levy (CFL) limit on the time-step size in a finite-difference time-domain (FDTD) simulation. In this new method, the periodic boundary conditions are implemented, permitting the simulation of a wide range of periodic optical media, i.e., gratings, or thin-film filters. Furthermore, the complete tensorial derivation for the permittivity also allows simulating anisotropic periodic media. Numerical results demonstrate that PITD is reliable and even considering anisotropic media can be competitive compared to traditional FDTD solutions. Furthermore, the maximum allowable time-step size has been demonstrated to be much larger than that of the CFL limit of the FDTD method, being a valuable tool in cases in which the steady-state requires a large number of time-steps.

## 1. Introduction

Simulation of the electromagnetic wave distribution through periodic media is a popular scenario in diffractive optics and photonics in general. Many optical devices are based on periodicity on at least one dimension (one-dimensionally periodic structures), and they can be used for different applications, i.e., waveguides, splitters, filters, etc. The analysis of this kind of element sometimes can be analysed by analytical closed expressions. However, in some applications, the fine detail of the structure is needed, thus implying a numerical formulation based on finite-element methods (FEM), FDTD or rigorous-coupled-wave theory (RCWT) amongst other numerical formulations. One of the most frequently used is FDTD due to its versatility and accuracy for electromagnetic analysis. However, the FDTD method has two main characteristics that limit the accuracy, stability, and indirectly the applicability of the method. These two factors are the Courant-Friedrich-Levy (CFL) condition and the dispersion error. Limiting the dispersion error implies considering small spatial resolutions compared to the wavelength, thus setting up very fine meshes. In these cases, the CFL condition forces small time-step sizes dramatically, thus resulting in demanding simulations in terms of running time and memory resources.

For improving the performance of FDTD, recently, a new approach for FDTD simulations has arisen. The PITD permits to break through the CFL limit in terms of time-step size. The basic idea follows the same paradigm as the traditional FDTD formulation, where the spatial derivatives are computed through the central finite-difference scheme. This step provides a set of ordinary differential equations (ODEs) that are solved by the precise integration (PI) [1,2]. In recent years, researchers have focused their efforts on taking advantage of this paradigm in different areas using the precise-integration time-domain (PITD) for solving Maxwell’s equations in free and lossy space [3,4,5,6,7,8]. There have been different contributions focused on improving PITD method: e.g., the extension of an unconditionally stable PITD for the numerical solutions of Maxwell’s equations to the circular cylindrical coordinate system was reported by Zhao et al. [9], and the works of Kang et al. related to the application of the PITD method to model the wave propagation in magnetised plasma based on the auxiliary differential equation (ADE) [10,11]. In the last years, some problems related to memory requirements on PITD have been addressed from different perspectives. Zhu et al. arranged the transverse electric/transverse magnetic (TE/TM) components in a matrix, constructing a set of Riccati matrix differential equations (RDEs). This proposal was able to reduce memory requirement compared to the conventional PITD for the analysis of homogenous media [12]. Shao et al. [13] implemented the so-called region-splitting (RS) technique for memory-saving realisations of the perfectly matching layers (PMLs) in the PITD method. However, even considering all these new approaches, the problems related to the memory requirements remain and, in some cases, limit the PITD method’s applicability. It is worth noting the recent contribution of Zhu et al. [14] in which a PITD method with a thresholding scheme is shown in order to reduce the computation cost of the matrix exponential involved on PITD.

To the best of our knowledge, the PITD method has not been explicitly applied to periodic optical media and neither to full anisotropic materials where the tensorial nature of the electrical permittivity is considered. The numerical analysis of periodical media through finite-difference approaches has been previously addressed in the literature. In diffractive optics, usually one-dimensional or two-dimensional structures are considered. Furthermore, there are some applications in which the optical device can have at least one dimension larger in terms of the input wavelength, e. g. holographic volume gratings [15,16]. Here, periodical boundary conditions help to minimise the simulation area, thus increasing the accuracy and performance of the numerical method. It is worth noting the outstanding contributions of Roden et al. [17] introducing a reliable formulation based on the split-field technique for simulating periodic structures at oblique incidence. The extension of SF-FDTD for simulating anisotropic media in 2D and 3D schemes was presented by Oh et al. [18,19], and Miskiewicz et al. [20,21], respectively. The authors have contributed to extending the SF-FDTD for the analysis of nonlinear optical media [22,23] and with the computational acceleration by means of graphic processing units (GPUs) [24]. There are other powerful approaches for the analysis of anisotropic optical media, i.e., finite element method (FEM) [25] or RCWA [26,27].

This work includes details related to the periodic boundary conditions (PBC) and anisotropic media implementation of PITD. In order to validate the approach, a set of well-known optical experiences are simulated, e.g., Young’s double-slit experiment, thin-film filters, dielectric binary diffraction gratings, and twisted-nematic liquid crystal (TNLC) regarding anisotropic optical media. The results show that the PITD method is accurate compared to split-field finite-difference time-domain (SF-FDTD) simulations. Furthermore, some analyses are performed related to the computational performance and also, the capability of using time-step resolution larger than the one established by the CFL condition is corroborated.

## 2. Formulation

As in the conventional FDTD method, Maxwell’s curl equations for inhomogenous and anisotropic media are expressed as

(1) $$\begin{array}{ccc} \nabla \times E & = & -\frac{\partial B}{\partial t} \end{array}$$

(2) $$\begin{array}{ccc} \nabla \times H & = & \frac{\partial D}{\partial t}+J \end{array}$$

(3) $$\begin{array}{ccc} \nabla \cdot D & = & \rho \end{array}$$

(4) $$\begin{array}{ccc} \nabla \cdot B & = & 0 \end{array}$$

The electric charge density is defined as ρ. The magnetic field H and the magnetic flux density B, as well as the electric field E and the electric flux density D, are connected via the following equations:

(5) $$\begin{array}{ccc} B & = & \mu H, \end{array}$$

(6) $$\begin{array}{ccc} D & = & \epsilon E. \end{array}$$

The magnetic permeability μ and the electric permittivity ϵ are in the most general case time-dependent and imaginary second-order tensors. The electric current density J on Equation (2) is connected to Ohm’s law via:

(7) J=σE,

where σ is the electric conductivity. In order to avoid big differences in the magnitude of the electric and magnetic field, the following change of variable is performed:

(8) $$\hat{E}=\sqrt{\frac{\epsilon_{0}}{\mu_{0}}}E,$$

where $\epsilon_{0}$ and $\mu_{0}$ are the relative permittivity, and relative permeability, respectively. For simplifying notation, from now $E=\hat{E}$. Taking into account Equations (5) and (6), the new normalized electric field Equation (8) can be introduced into Equations (1)–(4) obtaining the following set of equations:

(9) $$\begin{array}{ccc} \frac{\partial H}{\partial t} & = & -\frac{c}{\mu_{r}}\nabla \times E \end{array}$$

(10) $$\begin{array}{ccc} \frac{\partial E}{\partial t} & = & \frac{c}{\epsilon_{r}}\nabla \times H-\sigma^{\prime }E, \end{array}$$

being $\sigma^{\prime }=\frac{c}{\epsilon_{r}}\sqrt{\frac{\mu_{0}}{\epsilon_{0}}}\sigma =\frac{1}{\epsilon_{0}\epsilon_{r}}\sigma$, and *c* the speed of waves in vacuum.

If the material has lineal dielectric properties, only three dielectric constants ($\epsilon_{1}$, $\epsilon_{2}$ and $\epsilon_{3}$) and three geometric angles (α, β, and γ) are necessary to specify the full tensor description of $\epsilon_{r}$:

(11) $$\epsilon_{r}=\left[\begin{array}{ccc} \epsilon_{xx} & \epsilon_{xy} & \epsilon_{xz}\\ \epsilon_{yx} & \epsilon_{yy} & \epsilon_{yz}\\ \epsilon_{zx} & \epsilon_{zy} & \epsilon_{zz} \end{array}\right]=B^{-1}\left(\alpha ,\beta ,\gamma \right)\left[\begin{array}{ccc} \epsilon_{1} & 0 & 0\\ 0 & \epsilon_{2} & 0\\ 0 & 0 & \epsilon_{3} \end{array}\right]B\left(\alpha ,\beta ,\gamma \right),$$

where B is the transformation matrix fully defined in Equation (3) in [18] and related to the Euler angles (α, β, and γ). Defining $\kappa =\epsilon_{r}^{-1}$ and $\nu =\mu_{r}^{-1}$, the Equations (9) and (10) can be redefined as the following set of equations:

(12) $$\begin{array}{ccc} \frac{\partial H}{\partial t} & = & -c\nu \nabla \times E \end{array}$$

(13) $$\begin{array}{ccc} \frac{\partial E}{\partial t} & = & c\kappa \nabla \times H-\sigma^{\prime }E \end{array}$$

We limit ourselves to the analysis of 2D structures, but it is worth noting that applying this scheme to 3D problems would be done straightforwardly. If the simulation plane is in the xy plane, Equations (12) and (13) can be extended developing both curl terms in the right side and neglecting the spatial derivatives along *z*-axis.

(14) $$\begin{array}{ccc} \frac{dH_{x}}{dt} & = & c\nu_{xy}\frac{\partial E_{z}}{\partial x}-c\nu_{xx}\frac{\partial E_{z}}{\partial y}-c\nu_{xz}\left(\frac{\partial E_{y}}{\partial x}-\frac{\partial E_{x}}{\partial y}\right), \end{array}$$

(15) $$\begin{array}{ccc} \frac{dH_{y}}{dt} & = & c\nu_{yy}\frac{\partial E_{z}}{\partial x}-c\nu_{yx}\frac{\partial E_{z}}{\partial y}-c\nu_{yz}\left(\frac{\partial E_{y}}{\partial x}-\frac{\partial E_{x}}{\partial y}\right), \end{array}$$

(16) $$\begin{array}{ccc} \frac{dH_{z}}{dt} & = & c\nu_{zy}\frac{\partial E_{z}}{\partial x}-c\nu_{zx}\frac{\partial E_{z}}{\partial y}-c\nu_{zz}\left(\frac{\partial E_{y}}{\partial x}-\frac{\partial E_{x}}{\partial y}\right), \end{array}$$

(17) $$\begin{array}{ccc} \frac{dE_{x}}{dt} & = & c\kappa_{xz}\left(\frac{\partial H_{y}}{\partial x}-\frac{\partial H_{x}}{\partial y}\right)-\sigma_{xy}E_{y}-\sigma_{xz}E_{z}-\sigma_{xx}E_{x}-c\kappa_{xy}\frac{\partial H_{z}}{\partial x}+c\kappa_{xx}\frac{\partial H_{z}}{\partial y}, \end{array}$$

(18) $$\begin{array}{ccc} \frac{dE_{y}}{dt} & = & c\kappa_{yz}\left(\frac{\partial H_{y}}{\partial x}-\frac{\partial H_{x}}{\partial y}\right)-\sigma_{yy}E_{y}-\sigma_{yz}E_{z}-\sigma_{yx}E_{x}-c\kappa_{yy}\frac{\partial H_{z}}{\partial x}+c\kappa_{yx}\frac{\partial H_{z}}{\partial y}, \end{array}$$

(19) $$\begin{array}{ccc} \frac{dE_{z}}{dt} & = & c\kappa_{zz}\left(\frac{\partial H_{y}}{\partial x}-\frac{\partial H_{x}}{\partial y}\right)-\sigma_{zy}E_{y}-\sigma_{zz}E_{z}-\sigma_{zx}E_{x}-c\kappa_{zy}\frac{\partial H_{z}}{\partial x}+c\kappa_{zx}\frac{\partial H_{z}}{\partial y}, \end{array}$$

where $\kappa_{k,p}$, $\nu_{k,p}$ and and $\sigma_{k,p}$ are the components of the tensor k, ν and $\sigma^{\prime }$, with k,p=x,y, and *z*, respectively. For solving Maxwell’s equations in FDTD simulations the different electromagnetic field components are staggered in a computational grid space known as Yee’s cell [28,29]. The discrete expressions for Equations (14)–(16) can be written as:

(20) $$\begin{array}{ccc} \frac{dH_{x}{|}_{i+1/2,j}}{dt} & = & \frac{c\nu_{xz}}{2}\left(\frac{E_{y}{{|}_{i-1/2,j}-E_{y}|}_{i+3/2,j}}{\Delta x}\right.\\ & & \left.+\frac{E_{x}{|}_{i,j+1/2}-E_{x}{|}_{i,j-1/2}+E_{x}{{|}_{i+1,j+1/2}-E_{x}|}_{i+1,j-1/2}}{\Delta y}\right)\\ & & -\frac{c\nu_{xy}}{4\Delta x}\left(E_{z}{{|}_{i-1/2,j+1/2}-E_{z}|}_{i+3/2,j+1/2}\right.\\ & & \left.+E_{z}{{|}_{i-1/2,j-1/2}-E_{z}|}_{i+3/2,j-1/2}\right)\\ & & -\frac{c\nu_{xx}}{\Delta y}\left(E_{z}{{|}_{i+1/2,j+1/2}-E_{z}|}_{i+1/2,j-1/2}\right), \end{array}$$

(21) $$\begin{array}{ccc} \frac{dH_{y}{|}_{i,j+1/2}}{dt} & = & \frac{c\nu_{yz}}{2}\left(\frac{E_{x}{{|}_{i,j+3/2}-E_{x}|}_{i,j-1/2}}{\Delta y}\right.\\ & & \left.-\frac{E_{y}{|}_{i+1/2,j}+E_{y}{|}_{i+1/2,j+1}-E_{y}{{|}_{i-1/2,j}-E_{y}|}_{i-1/2,j+1}}{\Delta x}\right)\\ & & +\frac{c\nu_{yx}}{4\Delta y}\left(E_{z}{{|}_{i-1/2,j+1/2}-E_{z}|}_{i+3/2,j+1/2}\right.\\ & & \left.+E_{z}{{|}_{i-1/2,j-1/2}-E_{z}|}_{i+3/2,j-1/2}\right)\\ & & +\frac{c\nu_{yy}}{\Delta x}\left(E_{z}{{|}_{i+1/2,j+1/2}-E_{z}|}_{i-1/2,j+1/2}\right), \end{array}$$

(22) $$\begin{array}{ccc} \frac{dH_{z}{|}_{i+1/2,j+1/2}}{dt} & = & c\nu_{zz}\left(\frac{E_{x}{{|}_{i,j+1/2}-E_{x}|}_{i,j-1/2}}{\Delta y}-\frac{E_{y}{{|}_{i+1/2,j}-E_{y}|}_{i-1/2,j}}{\Delta x}\right)\\ & & +\frac{c\nu_{zy}}{2\Delta x}\left(E_{z}{{|}_{i+1/2,j+1/2}+E_{z}|}_{i+1/2,j-1/2}\right.\\ & & \left.-E_{z}{{|}_{i-1/2,j+1/2}-E_{z}|}_{i-1/2,j-1/2}\right)\\ & & -\frac{c\nu_{zx}}{2\Delta y}\left(E_{z}{{|}_{i+1/2,j+1/2}-E_{z}|}_{i+1/2,j-1/2}\right.\\ & & \left.+E_{z}{{|}_{i-1/2,j+1/2}-E_{z}|}_{i-1/2,j-1/2}\right). \end{array}$$

The unit cell has Δx, and Δy sizes. Note that employing a discrete grid implies that some values are not available directly, and hence they must be interpolated from neighbouring grids [18,30]. These additional approximations still hold the second order of accuracy. Note that each component is located in an auxiliary grid-point inside the Yee cell, i.e., $H_{y}$ is defined in (i,j+1/2). Hence, the spatial first-order partial differentiation on the right side of Equation (22) must be computed on (i,j+1/2) needing elements outside the grid cell in the grid boundary region. Considering normal incidence, the periodic boundary condition (PBC) scheme is

(23) $$\begin{array}{ccc} E(x=0,y) & = & E(x=\Delta ,y), \end{array}$$

(24) $$\begin{array}{ccc} H(x=0,y) & = & H(x=\Delta ,y), \end{array}$$

where Δ is the periodicity of the system. The same relationships can be applied for periodical structures along the *y*-axis. PBC is applied along the periodical axis for one-dimensional periodic structures, whereas perfectly matched layers (PML) are applied along the propagation direction. The implementation of PML for the PITD method is fully detailed in [13].

The discrete expressions for Equations (17)–(19) can be also written as:

(25) $$\begin{array}{ccc} \frac{dE_{x}{|}_{i,j+1/2}}{dt} & = & \frac{c\kappa_{xz}}{2}\left(\frac{H_{y}{{|}_{i+1,j+1/2}-H_{y}|}_{i-1,j+1/2}}{\Delta x}\right.\\ & & \left.+\frac{H_{x}{|}_{i+1/2,j}-H_{x}{|}_{i+1/2,j+1}+H_{x}{{|}_{i-1/2,j}-H_{x}|}_{i-1/2,j+1}}{\Delta y}\right)\\ & & -\frac{\sigma_{xy}}{2}\left(E_{y}{|}_{i+1/2,j}+E_{y}{|}_{i+1/2,j+1}+E_{y}{{|}_{i-1/2,j}+E_{y}|}_{i-1/2,j+1}\right)\\ & & -\sigma_{xz}\left(\frac{E_{z}{|}_{i+1/2,j+1/2}}{2}+\frac{E_{z}{|}_{i-1/2,j+1/2}}{2}\right)-E_{x}{|}_{i,j+1/2}\sigma_{xx}\\ & & -\frac{c\kappa_{xy}}{4\Delta x}\left(H_{z}{|}_{i+1,j}+H_{z}{|}_{i+1,j+1}-H_{z}{{|}_{i-1,j}-H_{z}|}_{i-1,j+1}\right)\\ & & -\frac{c\kappa_{xx}}{\Delta y}\left(H_{z}{{|}_{i,j}-H_{z}|}_{i,j+1}\right) \end{array}$$

(26) $$\begin{array}{ccc} \frac{dE_{y}{|}_{i+1/2,j}}{dt} & = & \frac{c\kappa_{yx}}{4\Delta y}\left(H_{z}{|}_{i,j+1}-H_{z}{|}_{i,j-2}+H_{z}{{|}_{i+1,j+1}-H_{z}|}_{i+1,j-1/2}\right)\\ & & -\frac{\sigma_{yx}}{4}\left(E_{x}{|}_{i,j+1/2}+E_{x}{|}_{i,j-1/2}+E_{x}{{|}_{i+1,j+1/2}+E_{x}|}_{i+1,j-1/2}\right)\\ & & -\frac{\sigma_{yz}}{2}\left(E_{z}{{|}_{i+1/2,j+1/2}+E_{z}|}_{i+1/2,j-1/2}\right)\\ & & -\frac{c\kappa_{yz}}{2}\left(\frac{H_{x}{{|}_{i+1/2,j+1}-H_{x}|}_{i+1/2,j-1}}{\Delta y}\right.\\ & & \left.+\frac{H_{y}{|}_{i,j+1/2}+H_{y}{|}_{i,j-1/2}-H_{y}{{|}_{i+1,j+1/2}-H_{y}|}_{i+1,j-1/2}}{\Delta x}\right)\\ & & -E_{y}{|}_{i+1/2,j}\sigma_{yy}+\frac{c\kappa_{yy}}{\Delta x}\left(H_{z}{{|}_{i,j}-H_{z}|}_{i+1,j}\right) \end{array}$$

(27) $$\begin{array}{ccc} \frac{dE_{z}{|}_{i+1/2,j+1/2}}{dt} & = & c\kappa_{zz}\left(\frac{H_{x}{{|}_{i+1/2,j}-H_{x}|}_{i+1/2,j+1}}{\Delta y}-\frac{H_{y}{{|}_{i,j+1/2}-H_{y}|}_{i+1,j+1/2}}{\Delta x}\right)\\ & & -\frac{\sigma_{zx}}{2}\left(E_{x}{{|}_{i,j+1/2}+E_{x}|}_{i+1,j+1/2}\right)-\frac{\sigma_{zy}}{2}\left(E_{y}{{|}_{i+1/2,j}+E_{y}|}_{i+1/2,j+1}\right)\\ & & -E_{z}{|}_{i+1/2,j+1/2}\sigma_{zz}+\frac{c\kappa_{zy}}{2\Delta x}\left(H_{z}{|}_{i,j}+H_{z}{|}_{i,j+1}-H_{z}{{|}_{i+1,j}-H_{z}|}_{i+1,j+1}\right)\\ & & -\frac{c\kappa_{zx}}{2\Delta y}\left(H_{z}{|}_{i,j}-H_{z}{|}_{i,j+1}+H_{z}{{|}_{i+1,j}-H_{z}|}_{i+1,j+1}\right) \end{array}$$

The above ODEs can be rewritten as a matrix form:

(28) $$\frac{dX}{dt}=MX+f\left(t\right),$$

where $X={\left(H_{x},H_{y},H_{z},E_{x},E_{y},E_{z}\right)}^{T}$ is a one-column vector containing both all of the electromagnetic field components. The matrix M contains the information related to the spatial-step and the medium parameters, and f(t) is a column vector with the excitations [3,10]. Here, harmonic plane waves are considered for illuminating the PITD grid. More specifically, a source line is placed parallel to the input plane of the device. This scheme is the same that the one used in standard FDTD simulations, e.g., [18]. The full derivation of the excitation source for polarized plane waves can be found in [31]. PBC is applied in Equations (14)–(19) over the unknown terms next to the boundary (out of the PITD grid). These terms are needed due to the spatial interpolation or the spatial derivative. It is worth mentioning that this paradigm is quite different to the one used in the traditional FDTD scheme, where Equations (23) and (24) can be directly applied after the updating of each electromagnetic field thanks to the leapfrog algorithm [28,29]. Here, this procedure can not be applied since all the components are computed at once when Equation (28) is solved.

Taking into account the theory of ODEs, the analytical solution of Equation (28) can be written as follows.

(29) $$X\left(t\right)=e^{Mt}X\left(0\right)+\int_{0}^{t}e^{M(t-s)}f\left(s\right)ds,$$

and the discrete from of this equation is

(30) $$\begin{array}{c} X_{k+1}=e^{Mt}X_{k}+T^{k+1}\int_{t_{k}}^{t_{k+1}}e^{-sM}f\left(s\right)ds, \end{array}$$

where $X_{k}=X\left(k\Delta t\right)$, Δt the time step, and $T=e^{M\Delta t}$ is the exponential matrix which can calculated by using the power rule:

(31) $$T={\left[e^{M\Delta t/2^{n}}\right]}^{2^{n}},$$

where *n* is a preselected arbitrary integer, such as n=20 [7,14]. An approximation of the term between brackets in Equation (31) is given as

(32) $$e^{M\Delta t/2^{n}}\approx I+T_{a},$$

where I is the identity matrix and $T_{a}$ is computed by the iterative process fully defined in Equations (5)–(7) in [14]. The interested reader in PI technique can find more specific information about the implementation in [7,10]. It is convenient to introduce here the Courant-Friedrichs-Lewy (CFL) condition for standard FDTD formulations to link it with the time step previously defined. The maximum time step in Yee’s FDTD is constrained by the CFL condition, which in 2D and square grid cell $\Delta_{x}=\Delta_{y}=\Delta$ reads

(33) $$s=\frac{c}{\sqrt{2}}\frac{\Delta t}{\Delta }\leq 1.$$

The CFL limit becomes particularly restrictive in the presence of small geometrical features, which impose a small cell size Δ and, consequently, a small-time step, leading to long simulations. Here, a smaller value for CFL condition is established as a reference for ensuring stability, so we define $\Delta t_{0}=\Delta /c$. PITD permits overcoming this CFL condition, and the syntax for addressing this feature is defined through the scaling factor $\alpha^{\prime }$ as $\Delta t=\alpha^{\prime }\Delta t_{0}$.

Returning to the PITD formulation, once T is known from Equations (31) and (32), the right-side of Equation (30) is approximated using the Gauss integration technique

(34) $$\begin{array}{ccc} X_{k+1} & = & {\mathrm{TX}}_{k}+\frac{5\Delta t}{18}\exp\left[\left(\frac{\Delta t}{2}+\frac{\Delta t}{2}\sqrt{\frac{3}{5}}\right)M\right]f\left(t_{k}+\frac{\Delta t}{2}-\frac{\Delta t}{2}\sqrt{\frac{3}{5}}\right)\\ & & +\frac{8\Delta t}{18}\exp\left[\frac{\Delta t}{2}M\right]f\left(t_{k}+\frac{\Delta t}{2}\right)\\ & & +\frac{5\Delta t}{18}\exp\left[\left(\frac{\Delta t}{2}-\frac{\Delta t}{2}\sqrt{\frac{3}{5}}\right)M\right]f\left(t_{k}+\frac{\Delta t}{2}+\frac{\Delta t}{2}\sqrt{\frac{5}{5}}\right) \end{array}$$

Once matrix M is fully defined, a thresholding scheme is applied for the matrix exponential terms in $T_{a}$. This procedure is fully detailed in [14] where sparse matrices are used for storing the exponential matrices, and the following thresholding scheme is considered:

(35) $${\left(T_{a}\right)}_{i,j}=\left\{\begin{array}{c} 0\;\;\mathrm{si}\;\;|{\left(T_{a}\right)}_{i,j}|\leq \alpha \delta \\ {\left(T_{a}\right)}_{i,j}\;\;\mathrm{otherwise}\;\; \end{array}\right.$$

where the threshold value is δ and the maximum absolute value of all elements in $T_{a}$ is α. In practice, the selection of threshold value is not necessary if δ is experimentally determined previously through a set of preliminary simulation tests. δ can be set as a smaller value to achieve higher accuracy. It is worth noting that δ=0 implies no threshold scheme, hence considering values between $10^{-5}$ and $10^{-8}$ should provide good results in almost all analysis. It should be mentioned that Zhu et al. [14] reported the application of the implicitly restarted ARnoldi method (IRAM) method for dynamically determining the value of δ. However, this approach has not been considered here due to the computation time that implies on each simulation run. Moreover, the convergence of the IRAM method is sometimes compromised, which is not an optimal solution considering the setup here.

## 3. Results

The diffraction pattern produced by one single slit or two slits is a very popular experiment in Physics since it shows the wave behaviour of light and is an experience that reinforces the Huygens principle and the relationship between diffraction and interference in Physics. The diffraction efficiency can be easily obtained through analytical closed expressions. However, for simulating this experiment numerically, some add-ons must be included. First, it is worth noting that the diffraction efficiency pattern is measured in the Fraunhofer region. Simulating areas far from the slits in PITD would require a considerable grid cell arrangement being impossible to simulate. Therefore, the near-to-far field transformation [28,29,32] has been implemented for obtaining the irradiance pattern in far-field. This far-field distribution is computed from the near-field electromagnetic field close to the slits computed by the PITD simulation. The analysis of one single slit or even two slits is not strictly a periodic problem. Hence, in this first set of analyses, PBC is disabled. This setup serves as an initial validation of the implementation and benchmark for simulating a typical scenario in diffractive optics where the grid form factor is far from being square and large in one dimension compared to the input wavelength.

**Figure 1:**
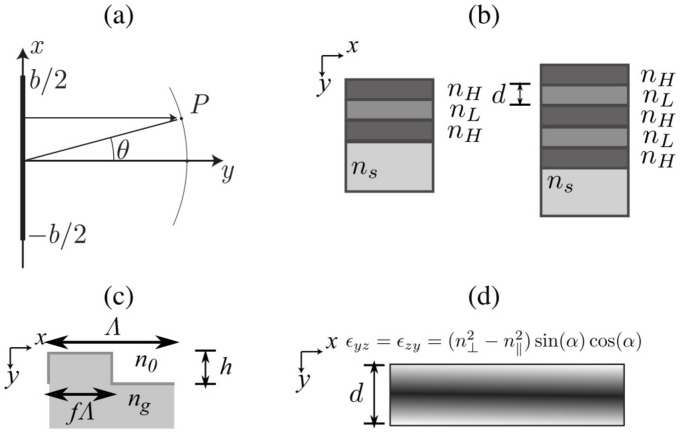
Schematic diagram of the media considered. (**a**) Scheme of a single slit of length *b*. (**b**) Two thin-film filters of 3 and 5 layers where high and low refractive indices are stacked. (**c**) Diffraction phase grating [33]. (**d**) Representation of the spatial variation of $\epsilon_{yz}$ and $\epsilon_{zy}$ in a TN-LC [18].

Figure 1a shows the scheme of a single slit of length *b*. θ is the angle formed between the normal of the slit and the path between the centre of the aperture and the observation point in the same plane.

**Figure 2:**
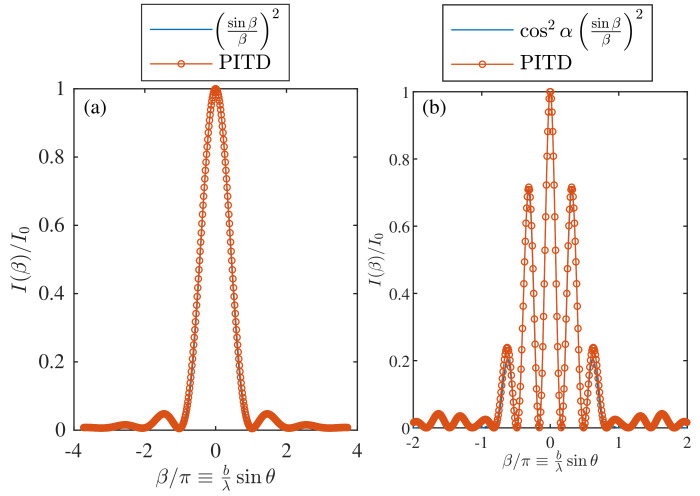
Simulation of the diffraction pattern of one and two slits of width *b* by means of PITD. The setup parameters are summarised in Table 1 (**a**) = *b* = 80Δ. (**b**) b=80Δ and a=150Δ.

Figure 2a shows the diffraction efficiency as a function of the parameter $\beta =\frac{kb}{2}\sin\theta$, where *b* is the length of the aperture, *k* is the wavenumber.

(36) $$I=I_{0}{\left(\frac{\sin\beta }{\beta }\right)}^{2},$$

where $I_{0}$ is the input light amplitude. For two slits, the Equation (36) can be reformulated with an additional term related to the separation of the slits *a* as:

(37) $$I=4I_{0}\cos^{2}\alpha {\left(\frac{\sin\beta }{\beta }\right)}^{2}.$$

where $\alpha =\frac{ka}{2}\sin\theta$. The results for two slits are shown in Figure 2b. The PITD setup is summarized in Table 1, where Δ and Δt are the spatial and time step resolutions, respectively. The parameter $\alpha^{\prime }$ is the factor applied to Δt to overcome the CFL condition. In this specific case, the relationship between Δ and Δt fits the CFL condition. The time window analysed is $t_{s}$, and the grid density (number of cells by wavelength) is defined as $N_{\lambda }$. The parameters $n_{y}$ and $n_{x}$ define the size of the simulation grid in cells for the *y* and *x* axis, respectively. Since the analysis of the diffraction pattern of slits is not a periodic problem, PBC is disabled only here, and PML in all boundaries of the simulation grid are considered. The number of additional cells considered for the absorbing boundary conditions is represented by $n_{\mathrm{PML}}$. The parameters *n* and δ are related to the PI formulation.

For modelling the metal wall that defines the aperture a considerably high value for $\sigma_{ii}=10^{7}$ S/F is considered in Equations (17)–(19). For the rest of parameters, $\nu_{ij}=0$ with i,j=x,y and *z*, since non-magnetic media is considered, and $\kappa_{ii}$ are defined as unity and $\kappa_{ij}=0$ with i≠j due to be the specific case in which homogeneous and isotropic media is considered.

**Figure 3:**
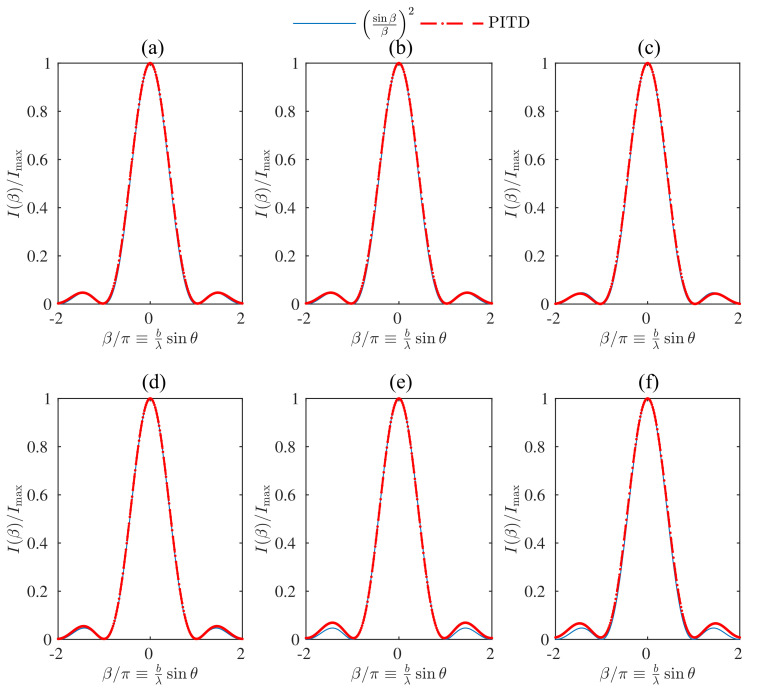
Simulation of the diffraction pattern of one slit of width b=80 cells by means of PITD. The setup parameters are summarised in Table 2. Each graph is related to a different $\alpha^{\prime }$ parameter: (**a**) $\alpha^{\prime }$ = 1. (**b**) $\alpha^{\prime }$ = 2. (**c**) $\alpha^{\prime }$ = 3. (**d**) $\alpha^{\prime }$ = 4. (**e**) $\alpha^{\prime }$ = 5. (**f**) $\alpha^{\prime }$ = 6.

Figure 3 shows the results of the simulation of one single slit but using a time-step resolution larger than the one established by the CFL condition. More precisely, $\alpha^{\prime }$ goes from one (CFL condition) up to six. The sequence Figure 3a–f shows the normalised irradiance as a function of the parameter β/π. It can be seen that the results are accurate in all cases. However, some discrepancies can be identified close to the second-order lobes for $\alpha^{\prime }\geq 5$. Hence, using larger values for $\alpha^{\prime }$ would not be recommendable due to the potential error obtained in that analysis and the drawbacks related to computational costs.

Table 2 summarises the parameters considered for the PITD simulation in Figure 3, whereas Table 3 shows some parameters related to the computational performance of this set of simulations. $N_{t}$ is the number of time-steps in order to simulate the 60 ns. As $\alpha^{\prime }$ rises, the number of time-steps is reduced. However, considering a constant threshold scheme implies that the size of T and $T_{i}$ also grows since increasing Δt implies larger values in these matrices; hence a larger number of values in this matrix are non-zero and are stored. The running time on Table 3 shows that even reducing the number of time-steps, the rise of the computation time of the matrices implies a significant increase in the total running time. This increase is mainly due to the overhead in the stage of setting up the matrices T and $T_{i}$ and in a lower degree, the impact that these structures induce in the time per iteration that also grows when $\alpha^{\prime }$ rises. Simulations performed in server with 2 Intel Xeon E5-2630-2.5 GHz, 15 MB Cache, 32 GB RAM DDR3 1333 MHz, SSD Samsung 840 Pro MZ-7PD512 6Gbps, and Mainboard Asus Z9PE-D8 WS and in MATLAB 2019b. Considering the threshold scheme shown in [14], where a variable δ parameter is computed is not worth the effort since the calculus of the eigenvalues needed for IRAM introduces a non-negligible penalty in terms of time simulation for the calculus of matrices T and $T_{i}$. Besides using IRAM, it has been experimentally proven that even considering to scale δ for each $\alpha^{\prime }$ scaling simulation, the overhead introduced by the growth of the memory previously detailed is not compensated. That is why a constant $\delta =10^{-5}$ parameter that has been previously determined is a more convenient strategy considering the setup here proposed. For comparing the results in Table 3 a FDTD simulation is performed with an equivalent setup of Table 1. Therefore, the traditional FDTD simulation takes 12.29 s for running a complete simulation with the finest time step for the same grid size (and the same number of time steps). The memory required for storing the simulation is 4.23 MB, and the time per iteration ratio is 9.7. As it can be affirmed, the traditional FDTD scheme is still more competitive than PITD, even the ability of PITD of overcoming the CFL condition considering this setup.

Figure 1b shows the scheme of the thin film filters (TFF) considered. Specifically, high-reflective coatings (HRCs) are is a basic type of TFF and is composed of a stack of alternate high- and low-index films, all one-quarter wavelength (considering $\lambda_{0}$ as the design wavelength for the structure) thick as it has been illustrated in Figure 1b. Light reflected within the high-index layers does not suffer any phase shift while a change of 180${}^{{}^{\circ}}$ in the low-index layers is produced [34]. It is straightforward to see that the light (with $\lambda =\lambda_{0}$) produced by reflection at successive boundaries throughout the assembly reappear at the front surface, all in phase so that they recombine constructively.

**Figure 4:**
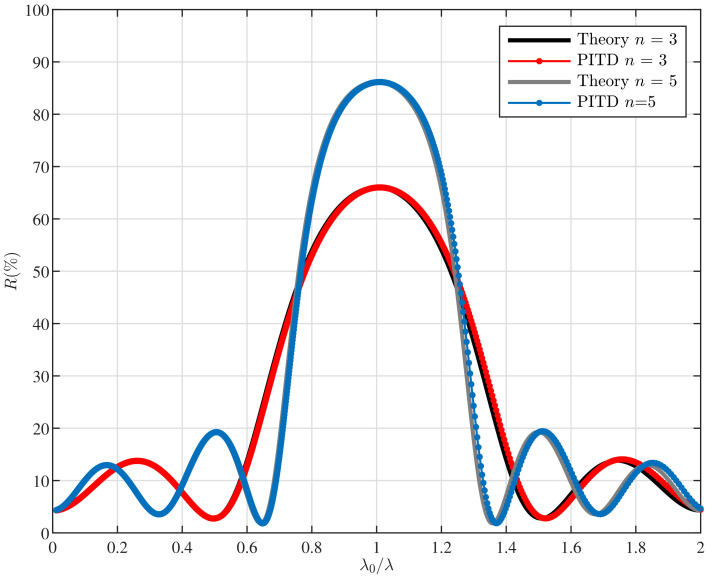
Simulation of the reflectance for two thin-film filters based on three layers and five stacks of layers. The setup parameters are summarised in Table 4.

Figure 4 shows the results obtained from PITD simulations and the theoretical curves by means of the matrix method fully detailed in [34]. The results show a good accuracy between the two methods considered. The PITD parameters setup are summarised in Table 4. The physical parameters for the refractive indices for the high and low layers arr $n_{H}$ = 2.3, and $n_{L}$ = 1.38, respectively. The refractive index of the substrate is $n_{s}$ = 1.52, and the excitation wavelength is $\lambda_{0}$ = 230 nm. It is worth mentioning that results show in Figure 4 are computed using $\alpha^{\prime }=\sqrt{2}$ taking advantage of the PITD capabilities.

**Figure 5:**
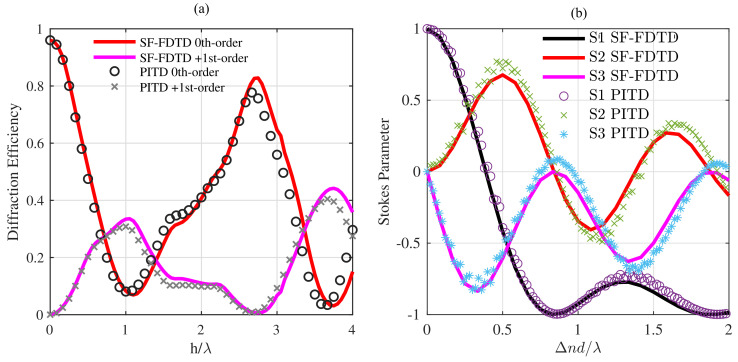
Comparison between PITD and SF-FDTD results of two periodic media. (**a**) Diffraction efficiency of the zeroth and first-order of a binary diffraction grating with period Λ=2λ, fill factor of 50% and refractive index of 1.5 [33]. SF-FDTD curve is reproduced with permission from Francés et al., In Proceedings of SPIE Optical Modelling and Design II, Vol. 8429, 2012. (**b**) Normalized Stokes parameters of a 90${}^{{}^{\circ}}$ twisted-nematic liquid crystal cell between with parameters: $\Delta n_{l}$ = 0.2, $n_{\|}$ = 1.5 [18].

Figure 5 shows the results of the PITD method applied to binary diffraction gratings and TNLC devices. More specifically, Figure 5a shows the diffraction efficiency of the 0th- and 1st-orders as a function of the normalised pillar length. The scheme of a binary phase grating is shown in Figure 1c where the parameters are the same ones used in the analysis in [33]: Δ=2λ, $n_{g}=1.5$ and f=50%. The length *h* has been varied for obtaining the results shown in Figure 5a. PITD simulation is compared to SF-FDTD results [33], showing that PITD provides close values to SF-FDTD. Some minor deviations are in the curves that can be produced by the spatial average that must be carried on in time-domain simulations and differences between spatial and time resolutions in both methods. Table 5 summarises the PITD setup for simulating the results in Figure 5a.

To demonstrate the potential of the PITD implementation here proposed an anisotropic device is simulated. More precisely, a TN-LC layer is considered. The scheme of this device is shown in Figure 1d. A TN-LC layer is based on LC layer of thickness *d* that has anisotropy along the *y*-axis (propagation direction) defined in Equation (38). The TNLC here considered is fully defined in section 3.1 in [18], being $n_{\|}=1.7$, $n_{⊥}=1.5$, $\Delta n=n_{\|}-n_{⊥}=0.2$ and $\phi_{\mathrm{twist}}=90^{{}^{\circ}}$. Therefore the $\epsilon_{r}$ matrix is defined as follows:

(38) $$\epsilon_{r}=\left[\begin{array}{ccc} n_{⊥}^{2} & 0 & 0\\ 0 & n_{⊥}^{2}\cos{\left(\alpha \right)}^{2}+n_{\|}\sin{\left(\alpha \right)}^{2} & \left(n_{⊥}^{2}-n_{\|}^{2}\right)*\sin\left(\alpha \right)\cos\left(\alpha \right)\\ 0 & \left(n_{⊥}^{2}-n_{\|}^{2}\right)*\sin\left(\alpha \right)\cos\left(\alpha \right) & n_{⊥}^{2}*\sin{\left(\alpha \right)}^{2}+n_{\|}^{2}*\cos{\left(\alpha \right)}^{2} \end{array}\right],$$

with $\alpha =\left(y/h\right)\phi_{\mathrm{twist}}$, being *h* the thickness of the TNLC layer. Interested reader into the details of the binary phase grating and the TNLC device here considered can find more specific information into [18,33], respectively.

Figure 5b shows the Stokes parameters obtained in the output plane of the TN-LC layer. In both cases (Figure 5a,b), the input light is linearly polarised along the *x*-axis with a wavelength λ = 633 nm.

Figure 5b shows the calculated polarization state of outgoing light from the TNLC cell in terms of the normalized Stokes parameters. Table 6 summarises the PITD setup for simulating the results in Figure 5b. The PITD results are compared with those obtained through SF-FDTD simulations and show a good agreement. It is worth noting that the results are consistent with those shown in Figure 3a in [18]. The differences between PITD here are related to the time-step resolution since the computation of Stokes parameter implies the estimation of the phase difference between electric field components. The resolution of the phase difference is closely related to the time resolution. In SF-FDTD, the Stokes parameter was directly related to the inner electric field components since the phase term is implicitly included in the split-field formulation. In spite of this PITD shows a good agreement in this case.

## 4. Conclusions

In this paper, the implementation of the PITD method for the analysis of periodic optical media is shown. More precisely, the formulation can simulate anisotropic periodic optical media. The scheme is fully defined, and some canonical examples are simulated and compared with the SF-FDTD method. PITD for optical media is validated through the diffraction efficiency computation of a single and two slits. Here, PITD is tested with time-step resolutions larger than the one established by the CFL condition. The stability of the method and accuracy is good enough for values of the time-step larger than six times than the largest time-step that fits with the CFL condition. A binary phase grating is also analysed regarding periodic optical media, obtaining good results compared to the SF-FDTD curves. Anisotropic periodic media is also covered through the analysis of a 90° TNLC layer. Here, the output polarisation state is analysed and compared to the ones obtained in the literature, showing that PITD is a good approach for simulating this type of devices. It is worth noting that simulating optical media where at least one or two dimensions are larger than the input wavelength can be afforded in the current state without any problems in terms of computational resources.

PITD has shown good accuracy for the analysis of periodic optical media. However, the method still has some drawnbacks related to the formulation. Even using sparse matrices, the memory requirements for time-steps larger than the one established by the CFL condition imply larger values for the matrices, and an experimental tuning of the threshold scheme must be carried out to minimise the overload in terms of computation time and memory requirements. Nevertheless, the authors consider that PITD can become a reference for the analysis of electromagnetic and optical materials where a fine mesh is needed, and hence larger time steps can be applied to reach the steady-state of the structure faster. However, more research and improvements must be developed to outcome the disadvantages that currently has this formulation.

## Figures and Tables

**Table 1:** Simulation parameters for Figure 2. $n_{x}=150$ for analysis in Figure 2a and $n_{x}=250$ for the results shown in Figure 2b.

Δ	$\alpha^{\prime }$	$\Delta t=\alpha^{\prime }\Delta t_{0}$	$t_{s}$	$N_{\lambda }$	$n_{y}$	$n_{\mathrm{PML}}$	*n*	δ
10 nm	1	$4.71\times 10^{-8}$ ns	20 ns	15	150	10	20	$10^{-5}$

**Table 2:** Simulation parameters for Figure 3.

Δ	$\alpha^{\prime }$	$\Delta t=\alpha^{\prime }\Delta t_{0}$	$t_{s}$	$N_{\lambda }$	$n_{y}$	$n_{x}$	$n_{\mathrm{PML}}$	*n*	δ
10 nm	$\sqrt{2}$	$4.71\times 10^{-8}$ ns	60 ns	60	100	600	30	20	$10^{-5}$

**Table 3:** Running time and memory resources of simulations shown in Figure 3.

$\alpha^{\prime }$	$N_{t}$	Size of T (MB)	Running Time T and $T_{i}$ (s)	Running Time PITD (s)	Time/Iter PITD (s)
1	1273	761.54	123.04	417.99	0.23
2	637	1594.44	236.59	519.13	0.44
3	425	2682.50	365.78	682.88	0.75
4	319	3960.6	584.55	915.78	1.04
5	255	5352.6	1090.52	1536.29	1.75
6	213	6889.0	1693.62	2151.43	2.15

**Table 4:** Simulation parameters for Figure 4.

Δ	$\alpha^{\prime }$	$\Delta t=\alpha^{\prime }\Delta t_{0}$	$t_{s}$	$N_{\lambda }$	$n_{y}$	$n_{x}$	$n_{\mathrm{PML}}$	*n*	δ
1.15 nm	$\sqrt{2}$	$5.42\times 10^{-9}$ ns	$10^{-8}$ ns	200	500	20	20	20	$10^{-5}$

**Table 5:** Simulation parameters for Figure 5a.

Δ	$\alpha^{\prime }$	$\Delta t=\alpha^{\prime }\Delta t_{0}$	$t_{s}$	$N_{\lambda }$	$n_{y}$	$n_{x}$	$n_{\mathrm{PML}}$	*n*	δ
52.7 nm	1	$87.9\times 10^{-9}$ ns	$125\cdot 10^{-9}$ s	12	120	24	10	20	$10^{-5}$

**Table 6:** Simulation parameters for Figure 5b.

Δ	$\alpha^{\prime }$	$\Delta t=\alpha^{\prime }\Delta t_{0}$	$t_{s}$	$N_{\lambda }$	$n_{y}$	$n_{x}$	$n_{\mathrm{PML}}$	*n*	δ
25 nm	1	$41.8\times 10^{-9}$ ns	$125\cdot 10^{-9}$ s	20	260	12	20	20	$10^{-5}$

## Data Availability

The data presented in this study are available on request from the corresponding author.
